# Cellular Senescence and Aging: Mechanisms, Disease Convergence, and Therapeutic Frontiers

**DOI:** 10.1002/mco2.70827

**Published:** 2026-07-05

**Authors:** Guowei Cai, Jia Ren, Li Wang, Yingying Wu, Huirui Wang, Xiaomeng Cao, Hao Shang, Xuben Hou, Yujiu Wang, Haibo Xue, Ting Dong

**Affiliations:** ^1^ Clinical Drug Trials Institution Binzhou Medical University Hospital Binzhou China; ^2^ State Key Laboratory of Bioactive Substance and Function of Natural Medicines Institute of Materia Medica Chinese Academy of Medical Sciences & Peking Union Medical College Beijing China; ^3^ Respiratory Medicine The Affiliated Hospital of Yan'an University Yan'an Shaanxi China; ^4^ College of Pharmaceutical Sciences Zhejiang University Hangzhou China; ^5^ Department of Natural Product Chemistry Key Laboratory of Chemical Biology (Ministry of Education) and State Key Laboratory of Discovery and Utilization of Functional Components in Traditional Chinese Medicine Shandong University Jinan Shandong China; ^6^ Department of Medicinal Chemistry State Key Laboratory of Discovery and Utilization of Functional Components in Traditional Chinese Medicine Shandong Key Laboratory of Druggability Optimization and Evaluation for Lead Compounds School of Pharmaceutical Sciences Cheeloo College of Medicine Shandong University Jinan Shandong China; ^7^ Department of Endocrinology and Metabolism Binzhou Medical University Hospital Binzhou China; ^8^ Institute of Medicinal Biotechnology Chinese Academy of Medical Sciences & Peking Union Medical College Beijing China

**Keywords:** aging hallmarks, age‐associated diseases, aging mechanisms, antiaging strategies, cellular senescence

## Abstract

Cellular senescence, a stress‐induced, irreversible cell cycle arrest coupled with a proinflammatory secretory phenotype, has emerged as both a central driver of aging and a tractable therapeutic target. Although acute senescence contributes beneficially to tumor suppression and wound healing, chronic accumulation of senescent cells sustains systemic inflammaging and accelerates organ dysfunction. This review synthesizes current progress in aging biology into a unified mechanistic and translational framework. We first examine how primary aging hallmarks, such as genomic instability and epigenetic dysregulation, interact to trigger cellular senescence, then explore how these converging molecular processes give rise to distinct age‐related pathologies across reproductive, pulmonary, hepatic, neurological, skeletal, and metabolic systems. We further evaluate emerging interventional strategies, from senolytics and senomorphics to rejuvenation approaches, while addressing key translational barriers including targeting specificity, senescence heterogeneity, and equitable access. By uniting mechanistic insight with disease‐oriented and therapeutic perspectives, this review charts a strategic roadmap for deploying senescence‐targeting therapies to extend human healthspan.

## Graphical abstract

This schematic illustrates the hierarchical and interconnected nature of the primary molecular hallmarks of aging. The progression of aging is driven by a convergence of intrinsic molecular insults. Within the nucleus, genomic instability and telomere attrition trigger persistent DDR, accompanied by extensive epigenetic alterations. In the cytoplasm, mitochondrial dysregulation acts as a major source of ROS, creating a vicious cycle of oxidative stress that further damages DNA and proteins. Concurrently, loss of proteostasis is manifested by the accumulation of misfolded proteins and the formation of dysfunctional SGs, a process exacerbated by macroautophagy dysfunction. Dysregulated nutrient signaling further disrupts cellular homeostasis. These cumulative intracellular damages culminate in the induction of cell senescence. The senescent cell secretes a SASP, releasing a cocktail of proinflammatory cytokines and proteases (depicted as red spheres). These factors propagate damage to neighboring healthy cells via paracrine signaling, driving chronic sterile inflammation known as inflammaging and accelerating systemic tissue dysfunction.

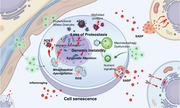

## 1 Introduction

The global demographic transition toward an older population poses mounting challenges for healthcare systems and socioeconomic structures alike. In China alone, the population aged 60 years and above surpassed 300 million by the end of 2024, a milestone that sharpens the urgency of advancing healthy aging strategies. Despite remarkable gains in life expectancy, these advances have not been matched by commensurate improvements in healthspan [1]. Over the past two decades, cellular senescence has emerged as a central mechanism underlying this disconnect. Senescence is defined by permanent cell cycle arrest and the senescence‐associated secretory phenotype (SASP), a heterogeneous cocktail of cytokines, chemokines, growth factors, and proteases [2]. Whereas acute senescence serves tumor‐suppressive and proregenerative functions, the progressive accumulation of senescent cells drives chronic inflammaging and diverse tissue pathologies, positioning senescence as a critical nexus between molecular damage and systemic physiological decline [3, 4, 5, 6, 7].

Despite considerable mechanistic progress, fundamental gaps persist in translating geroscience discoveries into clinical benefit. Research remains largely siloed within individual disease models, with insufficient integration across organ systems and molecular pathways. As a result, the associations among core aging hallmarks remain underexplored in the context of multimorbidity. Equally, the rapid expansion of senescence‐targeting therapeutics, though promising, has yet to be organized within a unifying framework linking disease‐specific biology to mechanism‐based intervention. This fragmentation hampers the rational design of combination therapies and companion biomarkers, underscoring the need for a cross‐disciplinary synthesis spanning mechanisms, disease convergence, and therapeutic innovation.

This review addresses these gaps through a systematic synthesis that transcends isolated observations to construct a cohesive aging framework. We begin by dissecting the core molecular hallmarks of aging, including genomic instability, epigenetic alterations, mitochondrial dysfunction, and proteostasis collapse, and delineating the positive feedback loops through which they reinforce one another. On this mechanistic foundation, we introduce the concept of disease convergence, systematically tracing how shared aging processes give rise to distinct clinical pathologies across six organ systems: reproductive, pulmonary, hepatic, neurological, skeletal, and metabolic. We then comprehensively survey therapeutic strategies organized by mechanism of action, from senolytics and senomorphics to emerging rejuvenation approaches, with a critical appraisal of clinical status. Through this integrated perspective, we aim to provide researchers and clinicians with a practical guide for converting fundamental geroscience into durable healthspan gains.

## 2 Molecular Hallmarks and Mechanisms of Aging

Organismal aging is not simply the cumulative wear and tear on individual cells or organs, but a systemic and progressive deterioration driven by the destabilization of core molecular networks. Decades of research have established that aging emerges from a set of conserved, deeply interconnected molecular and cellular hallmarks. This section examines the principal mechanisms underlying this complexity: how genomic instability and epigenetic dysregulation erode the fidelity of genetic information transmission and expression; how mitochondrial dysfunction and nutrient‐sensing dysregulation precipitate bioenergetic crisis and metabolic reprogramming; and how failure of proteostasis networks (PNs) drives the irreversible accumulation of toxic protein species. Critically, these processes do not act in isolation; they amplify one another through extensive crosstalk, collectively constituting the molecular substrate that drives cellular senescence. The ensuing accumulation of senescent cells then propagates age‐related disease at the tissue and organismal level, principally through SASP‐mediated paracrine signaling.

### 2.1 Genomic Instability

#### 2.1.1 Telomere Attrition

Telomeres are nucleoprotein complexes capping chromosome ends that buffer against replication‐associated sequence loss and protect chromosomal termini, thereby preserving genomic integrity [8]. With successive cell divisions and accumulating endogenous damage, particularly oxidative stress, telomeres erode to a critical threshold that triggers a p53‐centered DNA damage response (DDR), culminating in cell cycle arrest and senescence [9, 10]. Importantly, telomere dysfunction is not solely a product of length attrition: oxidative lesions such as 8‐oxoguanine within telomeric sequences can independently induce senescence without measurable shortening [11], underscoring the outsized sensitivity of these regions to redox damage. Critically shortened telomeres can also drive end‐to‐end chromosomal fusions, compounding genomic instability [12, 13], a mechanism operative in aged oocytes that directly compromises their quality and fertilization competence [14]. Clinically, telomere dysfunction is implicated in cancer [10] and metabolic syndrome [15], reinforcing its broad relevance to age‐related pathology. Therapeutically, telomerase delivery in a murine model of Hutchinson‐Gilford Progeria Syndrome (HGPS) attenuates DNA damage and inflammation while extending lifespan [16], illustrating the tractability of telomere‐targeted intervention.

#### 2.1.2 DNA Damage Accumulation

Genomic instability encompasses the full spectrum of heritable DNA alterations, from single‐nucleotide variants to large‐scale chromosomal rearrangements [17]. To counter this, cells deploy a tiered repair architecture comprising base excision repair, nucleotide excision repair, mismatch repair, nonhomologous end joining (NHEJ), and homologous recombination (HR) [18]. Nevertheless, chronic exposure to endogenous metabolic stress and exogenous genotoxic insults progressively overwhelms these defenses. Once DNA damage surpasses repair capacity, it establishes the molecular foundation of senescence [19]. Persistent lesions, including double‐strand breaks and aberrant secondary structures that stall transcription and replication, compromise genetic fidelity [17], while sustained ATM/ATR kinase signaling drives SASP release and disrupts tissue homeostasis [20, 21, 22]. The functional importance of robust repair is illustrated across multiple experimental contexts: loss of the neuronal NPAS4–NuA4 repair complex produces broad cellular defects and markedly shortened mouse lifespan [23]; HGPS cardiomyocytes accumulate extensive double‐strand breaks alongside impaired Progerin–γH2AX interaction and diminished NHEJ activity, accelerating cardiac atrophy [24]; and in naked mole‐rats, cGAS extends longevity by engaging HR repair proteins FANCI and RAD50 [25]. At the population level, whole‐genome sequencing of centenarians reveals globally elevated expression of DNA repair genes [26], and somatic mutation accumulation rates correlate inversely with species lifespan [27], collectively affirming DNA damage as a primary mediator of aging‐related deterioration.

### 2.2 Epigenetic Alterations

#### 2.2.1 Dysregulation of Epigenetic Modifications

Epigenetic modifications regulate gene expression and chromatin architecture without altering the underlying DNA sequence, collectively constituting a dynamic layer of genomic regulation [28]. Among these, DNA methylation remodeling and histone modification imbalances are most central to epigenetically driven senescence. Methylome erosion is a hallmark of both progeroid syndromes and physiological aging [29, 30]; promoter demethylation at *CDKN2A* directly induces senescence through INK4a/ARF upregulation [31], while pharmacological reversal of the age‐related methylation clock experimentally restores visual function in aged mice [32]. Beyond DNA methylation, histone modification landscapes shift comparably with age, featuring aberrant gains in H3K4 trimethylation and losses in H3K9/H3K27 trimethylation in both natural aging and progeroid contexts [28, 33, 34, 35]. The acetyltransferase KAT7, which catalyzes H3K14ac, is a positive regulator of senescence whose inhibition substantially extends healthspan [36]. Elevated histone lactylation, notably H3K18la and H4K12la, activates NF‐κB signaling and promotes vascular smooth muscle cell senescence, fueling SASP expression and atherosclerosis [37, 38], while H3K9 crotonylation is linked to renal fibrosis [39]. Epigenetic derepression in senescent cells additionally licenses transcription of endogenous retroviruses (ERVs), which engage the cGAS–STING innate immune axis to amplify senescence and inflammation through paracrine and humoral mechanisms [40], illustrating the breadth and dimensionality of epigenetic influence on organismal aging.

#### 2.2.2 Epigenetic Influence on Genomic Stability

Epigenetic regulation is deeply intertwined with the preservation of chromatin architecture, telomere integrity, and genome stability. Constitutive heterochromatin, a conserved feature of eukaryotic chromatin organization, is essential for transcriptional silencing of repetitive elements and maintenance of genomic order [41]. Aging erodes key repressive histone marks globally, precipitating widespread heterochromatin loss [28, 42] and permitting transcriptional reactivation of ERVs and other repetitive sequences [41], a direct route to genomic instability. Consistent with this, intestinal deletion of the H3K9 methyltransferase SETDB1 in mice triggers genomic instability and inflammation in the stem cell compartment [43], and erasure of centromeric DNA methylation causes chromosome missegregation and aneuploidy [44]. In humans, heterochromatin stability correlates positively with healthy aging in long‐lived individuals (LLIs) [45]. SIRT6 exemplifies the epigenome–genome stability axis: this histone deacetylase maintains telomeric heterochromatin, and its loss directly precipitates telomere dysfunction [46, 47, 48, 49]. Epigenetic regulation equally governs the DDR itself; chromatin relaxation at damage sites is a prerequisite for repair factor recruitment, precisely orchestrated through mechanisms including a deamidation‐acetylation cascade on linker histone H1 [50] and local recruitment of the metabolic enzyme PDHE1α to generate acetyl‐CoA for histone acetylation [51]. The recently characterized CHAMP1–POGZ–HP1α complex further coordinates genomic and telomeric stability by regulating heterochromatin condensates and HR repair [52]. Together, these findings position the epigenome as a frontline guardian against cell senescence and organismal aging through its indispensable role in genome maintenance.

### 2.3 Dysregulation of Metabolic Regulatory Systems

#### 2.3.1 Mitochondrial Dysfunction

Mitochondrial dysfunction, a canonical aging hallmark, manifests primarily as impaired oxidative phosphorylation and bioenergetic failure [53]. Mitochondria occupy a paradoxical position as both the principal source and a primary target of reactive oxygen species (ROS), generating a self‐amplifying damage cycle. ROS‐driven mitochondrial DNA (mtDNA) damage impairs respiratory chain complex function and provokes further bursts of superoxide [54]. The mitochondrial adaptor p66Shc exacerbates this loop by translocating to mitochondria to directly catalyze ROS production and trigger apoptosis [55, 56]; under hyperglycemic conditions, p66Shc additionally activates cGAS–STING signaling to drive senescence [57]. Compounding this, age‐associated depletion of the metabolic coenzyme NAD^+^—particularly within mitochondria—severely impairs biogenesis and antioxidant capacity [58, 59]. Damaged mitochondria release mtDNA into the cytoplasm, engaging cGAS–STING to sustain sterile inflammation and fuel inflammaging [60]. In the vasculature, this oxidative–metabolic crisis is especially consequential: excess superoxide rapidly quenches nitric oxide (NO), producing toxic peroxynitrite and driving endothelial dysfunction and arterial stiffening [61].

Mitochondrial homeostasis is safeguarded by an integrated quality control network encompassing the mitochondrial unfolded protein response (UPRmt) and mitophagy [62]. The UPRmt is an evolutionarily conserved stress program that restores mitochondrial proteostasis, maintains systemic metabolic balance, and shapes epigenetic landscapes through mitochondrial metabolism [63, 64, 65]. Recent work shows that UPRmt suppresses histone acetylation by reducing acetyl‐CoA via c‐Jun, thereby restraining the mesenchymal‐to‐epithelial transition and offering a potential lever for countering stem cell aging [65]. Loss of the mitochondrial protease ClpP in ClpP^−^^/^^−^ mice produces growth retardation, lethargy, and reduced survival [66], illustrating the essentiality of this proteostatic machinery. When damage is irreparable, mitophagy clears dysfunctional organelles through PINK1‐dependent ubiquitin signaling at the outer mitochondrial membrane [67, 68]. PINK1 deficiency impairs autophagy flux and accelerates kidney aging via sustained cGAS–STING activation [69], whereas restoring mitophagy with the inducer urolithin A clears cytosolic mtDNA, silences cGAS–STING, and alleviates aging‐related neurological dysfunction [70]. Beyond cell‐autonomous effects, neuronal mitochondrial damage can spread pathology to neighboring glia, inducing glial senescence and lipid accumulation that amplifies neuroinflammatory aging [71]. Given its convergence on oxidative stress, metabolic reprogramming, and innate immune activation, mitochondrial dysfunction is emerging as a central hub of systemic multimorbidity in cardiovascular disease (CVD), neurodegeneration, and metabolic syndrome [72, 73, 74], and one of the most compelling targets for antiaging intervention.

#### 2.3.2 Oxidative Stress

Both the free radical theory and the oxidative‐inflammation theory identify ROS as primary mediators of cellular aging [75, 76]. When antioxidant defenses are overwhelmed, oxidative damage accelerates telomere erosion and DNA double‐strand breaks [76]. This burden is compounded by exogenous stressors, radiation, high‐fat and high‐sugar diets, tobacco, alcohol, and environmental toxins, while endogenous antioxidant capacity simultaneously wanes with age [77]. The resulting chronic oxidative imbalance contributes to osteoarthritis (OA), osteoporosis (OP), and other age‐related conditions [78]. The redox regulator thioredoxin modulates oxidative stress‐driven cell senescence and systemic aging [79], and the neuroprotective protein DJ‐1 shields neurons from ROS‐induced damage, its loss accelerating retinal aging and heightening neuronal vulnerability in mice [75]. Beyond telomere shortening, mitochondria‐derived ROS can attack T cell telomeres directly, inducing fragility and DNA strand breaks [80]. Oxidative stress also impairs the translational machinery, generating misfolded proteins that overwhelm cytoplasmic and mitochondrial protein quality control networks [81, 82]. Oxidative stress thus functions not merely as an effector of cellular damage but as an integrating signaling node that amplifies and connects multiple parallel injury pathways.

#### 2.3.3 Dysregulation of Nutrient Sensing

The nutrient‐sensing network enables cells to balance growth and repair in response to resource availability, and its progressive dysregulation is a defining feature of metabolic aging. The mTOR and AMPK pathways form the core of this network, acting as a molecular switch through competitive phosphorylation of the autophagy‐initiating kinase ULK1: mTORC1 phosphorylates ULK1 under nutrient‐replete conditions to suppress autophagy, whereas glucose limitation activates AMPK to phosphorylate ULK1 and promote it [83]. As highly conserved nutrient‐sensing and growth coordination centers, mTOR and AMPK coordinately regulate protein synthesis [84, 85, 86], metabolism [87], autophagy [88, 89], and mitochondrial function [90, 91]. Pathological mTORC1 hyperactivation in mice drives bone marrow inflammation and shortens lifespan [92], while inhibition of the downstream effector S6K1 recapitulates caloric restriction (CR) transcriptomics and extends mammalian lifespan [93], an effect dependent on endolysosomal integrity in the fat body, where S6K hyperactivation distorts lysosomal morphology and triggers inflammaging [94]. AMPK extends lifespan through a distinct mechanism: phosphorylation of the nucleoporin NPP‐16/NUP50 enables it to act as a transcriptional coactivator of lipid metabolism genes, remodeling the lipid metabolic network [95].

The insulin/IGF‐1 signaling (IIS) pathway is equally central to aging regulation across species [96, 97, 98, 99]. Insulin‐like growth factor 1 (IGF1)‐activated Akt phosphorylates FOXO3a (FKHRL1), sequestering it in the cytoplasm and blocking transcription of proapoptotic targets [100]. While this supports growth, it simultaneously prevents FOXO3a from inducing antioxidant enzymes (e.g., MnSOD) and DNA repair genes, accelerating senescence through inadequate cellular maintenance [101, 102]. The spatial logic of IIS regulation has been clarified in *Caenorhabditis elegans* (*C. elegans*): intestine‐specific degradation of the IIS receptor DAF‐2 alone is sufficient to drive systemic metabolic remodeling resembling dietary restriction and to extend lifespan [97]. Age‐related IIS dysregulation also perturbs intestinal stem cell differentiation via the RAB7–SOX21A axis, impairing epithelial renewal and digestive homeostasis [103].

The gut microbiome functions as a critical exogenous nutrient sensor and has been aptly described as a hidden metabolic organ. It governs energy and glucolipid balance, immune surveillance, mucosal integrity, and xenobiotic metabolism [104]. Centenarians harbor a characteristically youthful microbiome enriched in Bacteroidetes and depleted of pathogens [105], an ecological signature mediated partly through bioactive metabolites, including short‐chain fatty acids [106]. Longevity‐associated secondary bile acids such as isoallo‐lithocholic acid (LCA) and the metabolite PTA2 confer potent antipathogenic and neuroprotective effects, respectively [107, 108]. Gut dysbiosis disrupts the gut–brain axis, exacerbating neurodegenerative progression [109], while the gut virome promotes sulfur metabolism in ways that may further support intestinal resilience [110]. Together, these findings establish the gut microbiome as a dual hub for nutrient sensing and intertissue signal transduction in systemic aging.

In studies of extreme longevity, the coupling of nutrient sensing to metabolism emerges as a defining feature of LLIs. LLI monocytes sustain exceptionally high insulin sensitivity, enabling nuclear insulin receptor‐driven gene regulation that preserves lysosomal clearance and immune youthfulness [111]. Strikingly, nutrient‐sensing signals can also propagate across generations: lipid signals from intestinal lysosomes facilitate trans‐organ transport of histone variant H3.3 to the germline, imprinting a specific H3K79 methylation pattern that transmits metabolically encoded longevity memory to offspring [112]. This conversion of transient metabolic cues into heritable epigenetic states reveals a deep mechanistic coupling between nutrient sensing and epigenetic regulation in aging, and opens the possibility that metabolic interventions may exert multigenerational effects.

### 2.4 Loss of Protein Homeostasis

#### 2.4.1 PN Failure

Proteostasis, the maintenance of proteins at appropriate concentrations and conformations through coordinated chaperone, degradation, and quality control systems, falters along two principal axes during aging [113, 114]. At the folding level, molecular chaperone efficacy declines with age, allowing misfolded proteins to accumulate [115, 116]. Many misfolded intermediates escape chaperone recognition due to surface properties resembling native protein, persisting in soluble yet dysfunctional states that silently erode cellular function [117, 118], a problem with particular severity in postmitotic cells such as cardiomyocytes, where such burdens translate directly into organ dysfunction [119]. At the degradation level, ubiquitin–proteasome system (UPS) activity progressively wanes [120], and the ubiquitination landscape of the aging brain shifts substantially, partly attributable to declining proteasome activity [121]. Posttranslational regulation adds further complexity: aberrant PINK1 kinase activity generates abnormal phosphorylation of ubiquitin at Ser65, a modification now recognized as a clinical biomarker of mitochondrial damage in neurodegenerative disease [120, 122]. Collectively, these deficits make the restoration of folding capacity and the enhancement of proteasomal clearance central priorities for countering age‐related proteostasis collapse.

#### 2.4.2 Macroautophagy Dysfunction

Among the three autophagic modalities, namely, macroautophagy, microautophagy, and chaperone‐mediated autophagy, macroautophagy (hereafter autophagy) is the dominant pathway for intracellular renewal [60], capable of engulfing proteins, lipids, damaged organelles, and pathogens within double‐membrane autophagosomes for lysosomal degradation [123]. With age, autophagic flux deteriorates across multiple steps: autophagy‐related protein expression falls, autophagosome formation slows, and lysosomal acidification and hydrolytic capacity decline [124, 125]. The downstream consequences are profound; damaged organelles and toxic protein aggregates accumulate, directly disrupting proteostasis and precipitating an energy crisis. This dysfunction is particularly consequential at the mitochondrial level, where impaired mitophagy allows defective mitochondria to persist and generate sustained ROS bursts that further destabilize lysosomal membranes, creating a self‐reinforcing deterioration of autolysosomal function [60]. Pathologically, autophagy dysfunction is critical in the progression of neurodegenerative diseases [123]. In neurons, whose proteostatic demands are especially stringent, autophagic failure permits the buildup of amyloid‐β (Aβ) and α‐synuclein (α‐Syn) aggregates, directly mediating neuronal death, amplifying oxidative stress, and igniting local inflammation [126]. Extensive animal model studies confirm a tight coupling between autophagic competence and lifespan [127], and autophagic decline is now recognized not merely as a senescence correlate but as an active driver of neurodegenerative disease, CVD, and cancer [128].

#### 2.4.3 Stress Granules

Stress granules (SGs) are dynamic, membraneless condensates that assemble in response to stressors, including oxidative insult and heat shock [129, 130], and their functional role in aging is inherently dual. Under acute stress, transient SG assembly constitutes a frontline defense for proteostasis and RNA homeostasis. SGs rapidly concentrate at sites of lysosomal membrane damage to physically prevent content leakage and facilitate membrane repair [131], sequester nonessential mRNAs and translation initiation factors to preserve cellular integrity [132], and create an internal reductive microenvironment that shields client proteins from oxidative degradation while fine‐tuning cytoplasmic redox signaling [133]. They additionally relieve pressure on the UPS by isolating misfolded proteins, suppressing stress‐induced mistranslation [134], and recruiting proteasomes to maintain compositional homeostasis [135], collectively bolstering survival and attenuating apoptosis under adverse conditions [132].

These protective functions, however, are contingent on dynamic assembly‐disassembly cycling. When stress is chronic or the disaggregation machinery is compromised, SGs accumulate pathologically, which leads to the collapse of proteostasis and persistent inflammation. In *C. elegans*, age‐dependent accumulation of core SG components PAB‐1 and TIAR‐2 directly correlates with shortened lifespan [136]. Aberrant SGs sequester key autophagic factors such as HDAC6, obstructing autophagic clearance [137]; impaired autophagy then fails to resolve these aberrant condensates, completing a vicious cycle that accelerates senescence and neurodegeneration. Notably, attenuated IIS signaling reduces pathological SG accumulation [138], underscoring the crosstalk between nutrient sensing and proteostasis. SG dynamics thus represent a critical switch between cellular resilience and pathology, and preserving their normal cycling is integral to healthy aging.

**FIGURE 1:**
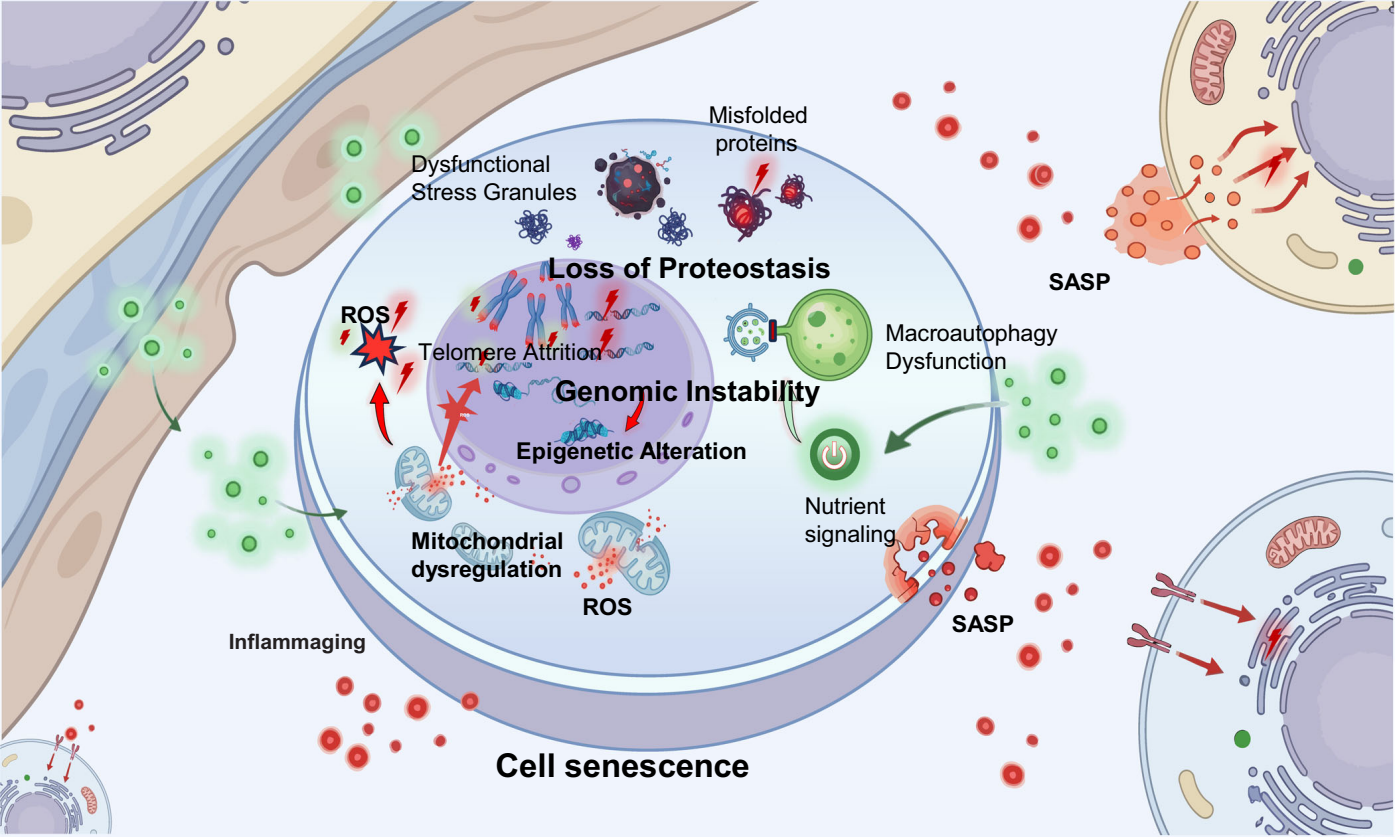
The integrated molecular network driving cellular senescence and systemic inflammaging. This schematic depicts the hierarchical and interconnected molecular hallmarks that collectively drive cellular senescence and systemic inflammaging. Within the nucleus, genomic instability and telomere attrition trigger persistent DDR, accompanied by extensive epigenetic dysregulation. In the cytoplasm, mitochondrial dysfunction generates ROS that damage DNA and proteins in a self‐amplifying cycle. At the same time, proteostasis collapse manifests as accumulation of misfolded proteins, dysfunctional stress granule formation, and failure of macroautophagy. Dysregulated nutrient sensing further erodes cellular homeostasis. These converging intracellular insults culminate in the induction of cellular senescence. Senescent cells, in turn, secrete the SASP, a proinflammatory cocktail of cytokines and proteases (depicted as red spheres), which propagates damage to neighboring cells via paracrine signaling, sustaining the chronic sterile inflammation known as inflammaging and accelerating tissue dysfunction across organ systems.

The molecular hallmarks of aging do not act in isolation; they form a tightly coupled, self‐amplifying network whose collective breakdown converges on cellular senescence (Figure 1). Irreparable DNA damage, persistent mitochondrial oxidative stress, and unresolved protein aggregates together trigger irreversible cell cycle arrest. Critically, senescent cells are not passive endpoints: through SASP secretion, they convert localized molecular damage into tissue‐wide inflammaging, propagating dysfunction from the cellular to the organ level [2, 7, 139]. This SASP‐driven microenvironmental disruption is the mechanistic bridge between microscopic lesions and macroscopic pathology. With senescence established as the integrating hub of these interconnected processes, the following section shifts focus from molecular origins to clinical consequences, examining how this shared mechanistic substrate drives convergent age‐related pathologies across organ systems.

## 3 Disease Convergence: Aging as the Common Ground

The Geroscience Hypothesis holds that the cellular and molecular changes of aging constitute a shared pathological substrate for virtually all chronic diseases [140]. As detailed above, genomic instability, epigenetic drift, and mitochondrial dysfunction converge on cellular senescence, which in turn bridges molecular insults to macroscopic pathology [3]. This convergence is strikingly evident across organ systems: age‐related endothelial dysfunction underlies CVD, including atherosclerosis and hypertension [141], while the combined collapse of proteostasis, mitochondrial integrity, and redox balance drives neurodegenerative conditions such as Alzheimer's disease (AD) [142]. Particularly compelling is the evolutionary paradox between aging and cancer; although senescence originally evolved as a tumor‐suppressive mechanism, the progressive accumulation of senescent cells and their SASP paradoxically constructs a protumorigenic microenvironment, making aging the single greatest cancer risk factor [143, 144].

**FIGURE 2:**
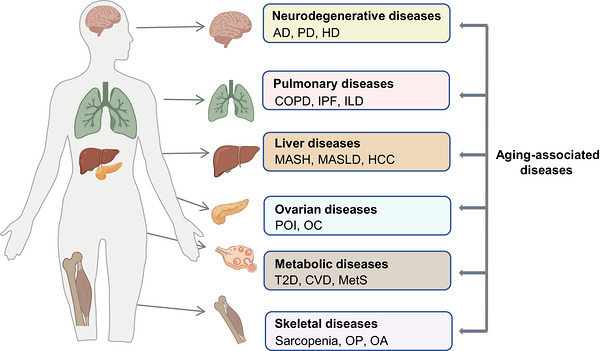
Systemic manifestations of aging‐associated diseases across major organ systems. This schematic illustrates how aging drives pathology across distinct physiological systems, underscoring the concept of aging‐associated multimorbidity. Rather than a singular event, aging manifests as a spectrum of organ‐specific diseases sharing common molecular origins, organized here into six domains: (1) neurodegenerative diseases, including Alzheimer's disease (AD), Parkinson's disease (PD), and Huntington's disease (HD); (2) pulmonary diseases, encompassing chronic obstructive pulmonary disease (COPD), idiopathic pulmonary fibrosis (IPF), and interstitial lung disease (ILD); (3) liver diseases, including metabolic dysfunction‐associated steatohepatitis (MASH), metabolic dysfunction‐associated steatotic liver disease (MASLD), and hepatocellular carcinoma (HCC); (4) ovarian diseases, specifically premature ovarian insufficiency (POI) and ovarian cancer (OC); (5) metabolic diseases, connecting type 2 diabetes (T2D), cardiovascular disease (CVD), and metabolic syndrome (MetS) as a systemic hub; (6) skeletal diseases characterized by regenerative failure, including sarcopenia, osteoporosis (OP), and osteoarthritis (OA). The diversity of these clinical manifestations reflects the systemic reach of the aging process.

With this mechanistic convergence in view, this section examines six representative categories of age‐related disease spanning distinct organ systems (Figure 2), each with well‐established causal ties to aging and evidence supporting aging‐targeted intervention. We begin with the reproductive system, where accelerated aging is most biologically conspicuous, then turn to chronic inflammation and fibrosis in the respiratory and hepatic systems. Proteostasis failure in the nervous system and regenerative collapse in the musculoskeletal system illustrate aging's impact on highly specialized and dynamically renewing tissues, respectively. Finally, metabolic syndrome exemplifies the transition from localized dysfunction to systemic multimorbidity, linking insulin resistance and CVD as the metabolic face of organismal aging. Viewed together, these diseases are not independent entities but tissue‐specific expressions of the same fundamental process, a reframing that reveals the transformative therapeutic potential of targeting aging at its root rather than managing its downstream manifestations.

### 3.1 Ovarian Diseases

Ovarian aging is defined by irreversible follicular depletion, declining oocyte quality, progressive fibrosis, and metabolic dysregulation, and pronounced hallmarks of aging are evident across multiple premature ovarian insufficiency (POI) models [145, 146, 147]. The initiating lesions arise within germ cells themselves: meiotic defects, eroding DNA repair capacity, and cumulative genomic damage collectively exhaust the ovarian follicle pool [145, 148, 149, 150, 151]. In aged mouse oocytes, several intrinsic deficits converge to compromise meiotic fidelity, mevalonate pathway dysregulation induces chromosome segregation errors [149], inefficient m^6^A‐mediated translation by YTHDF3 depletes key factors such as HELLS and impairs epigenetic fidelity and DNA repair [148], and age‐related actin dysfunction compounds aneuploidy arising from centromeric cohesion loss [152]. Simultaneously, granulosa cells deteriorate functionally, with heightened oxidative stress and diminished mitochondrial output, reducing their metabolic support for oocytes and establishing a self‐reinforcing cycle of ovarian decline [153]. At the tissue level, the ovarian stroma stiffens progressively with age, disrupting granulosa cell–oocyte communication through aberrant mechanotransduction and impairing secondary follicular development [154]. The resulting chronic inflammatory microenvironment elevates susceptibility to ovarian cancer (OC) [155, 156]. Intriguingly, genetic evidence reveals that rare variants in genes such as *SAMHD1* simultaneously delay menopause and increase multicancer risk, suggesting that ovarian aging and cancer susceptibility share common regulatory pathways rather than a simple cause‐and‐effect relationship where accelerated aging directly drives cancer [157]. In sum, converging upstream drivers, including genomic instability, epigenetic dysregulation, oxidative stress, granulosa cell mitochondrial dysfunction, and metabolic reprogramming, induce senescence and stromal stiffening that culminate in follicular depletion, oocyte deterioration, and a cancer‐permissive ovarian microenvironment.

### 3.2 Pulmonary Diseases

The incidence of chronic obstructive pulmonary disease (COPD), interstitial lung disease (ILD), and idiopathic pulmonary fibrosis (IPF) rises steeply with age, and their molecular signatures overlap substantially with aging biology; telomere attrition and genomic instability drive cellular senescence, which in turn mediates tissue pathology [158]. Senescence restructures lung architecture and cellular function to accelerate chronic respiratory disease progression [159]. In IPF, alveolar epithelial cells display elevated senescence markers p16 and p21 [160], and telomere dysfunction in alveolar type 2 (AT2) cells drives senescence‐associated extracellular matrix (ECM) remodeling that propagates fibrosis [161]. Elevated plasma fibronectin 1 increases IPF risk by accelerating telomere erosion, implicating senescence as a direct fibrotic driver [162]. Metabolic reprogramming amplifies these changes: propionate metabolism defects cause methylmalonic acid accumulation that activates profibrotic signaling [163], while epoxyeicosatrienoic acids counter alveolar epithelial senescence by suppressing endoplasmic reticulum (ER) stress through the NRF2 axis [164]. As in the ovary, perialveolar matrix stiffening activates fibroblasts and reinforces the fibrotic loop through integrin–FAK and YAP/TAZ mechanotransduction [165]. Beyond fibrosis, aging‐driven hematopoietic and immune dysregulation fosters a proinflammatory IL‐1α‐enriched microenvironment that promotes lung cancer development [166]. Accordingly, senescent cell clearance and modulation of aging‐related pathways have demonstrated efficacy in attenuating pulmonary fibrosis and inflammation (Table 1).

**TABLE 1:** Age‐related diseases: biomarkers, senescence/aging involvement, and therapeutic evidence.

Disease categories	Biomarkers	Aging/senescence involvement	Intervention evidence
Ovarian diseases	p16↑, p21↑ [145], NCOA7↓ [214]	Genomic instability: idiopathic POI patients harbor DNA repair defects [151]. Mitochondrial dysfunction and excessive AMPK activation impair meiotic progression [215]. Lysosomal dysfunction: NCOA7 deficiency impairs lysosomal acidification and accelerates senescence [214].	Spermidine activates AMPK–mTOR signaling, promotes mitophagy, and restores oocyte chromosome alignment [216]. Rapamycin induces autophagy, clears stress granules, and preserves ovarian function [214]. Baicalin enhances granulosa cell function via mTOR/Nrf2‐regulated autophagy and redox balance [217]. NCOA7 mRNA delivery restores lysosomal function and reverses senescence [214]. CD38 inhibitor 78c elevates ovarian NAD^+^, improves oocyte quality, and attenuates ovarian aging [146].
Pulmonary diseases	p16↑, p21↑, LAMP1 [218],	Senescent AT2 cells and fibroblasts secrete SASP, remodel the ECM, and drive fibrosis [219]. EETs metabolic dysfunction triggers ER stress [163].	Dasatinib + quercetin (D+Q) clears senescent AT2 cells in aged mouse lungs and halts fibrosis [220]. BTSA1 selectively induces apoptosis in senescent fibroblasts, reducing collagen deposition and attenuating lung injury [221]. Targeted p16^+^ senescent cell clearance reverses smoking‐induced mitochondrial dysfunction and inflammation while restoring lung architecture [222]. NAD^+^ supplementation lowers the airway inflammatory factor IL‐8 in COPD patients [223]. Aescin upregulates NPNT expression, reduces senescent epithelial cell burden, and improves lung function in pulmonary fibrosis models [219]. Cortex Mori Radicis (CMR) inhibits PI3K/Akt signaling and delays fibroblast senescence in COPD [224].
Liver disease	p16↑, p21↑, SIRT7↓ [225]	Metabolic dysregulation: the lipid peroxidation product 13‐HODE induces hepatocyte senescence via NETs [172]. Hedgehog pathway inhibition exacerbates hepatic senescence and MASH pathogenesis [226].	DpC, the first hepatocyte‐specific senolytic, selectively eliminates senescent liver cells and reverses fibrosis [169]. PROTAC 753b targets BCL‐xL/BCL‐2 to clear senescent cells and suppress tumor growth in a MASLD‐HCC model [227]. Nicotinamide (NAM) prevents aging and restores lipid metabolic balance in alcoholic liver disease [228]. CDK4/6 inhibitor PD‐0332991 induces senescence in tumor‐initiating cells, reducing HCC incidence and attenuating liver damage at the precancerous stage [229].
Metabolic disorders	IL‐6, TNF‐α↑ [210]	β‐Cell senescence: metabolic stress and aberrant receptor cleavage cause cell cycle arrest and loss of insulin secretion [207]. Inflammatory environment: senescent β‐cells secrete IL‐6/TNF‐α, activating distal fibroblasts and worsening insulin resistance and complications [210]. Adipose tissue aging: adipocyte hypertrophy with immune cell infiltration drives proinflammatory macrophage polarization and systemic insulin resistance [200]. Endothelial senescence impairs NO synthesis and promotes atherosclerosis [202].	ABT263 clears senescent cells and accelerates wound healing in diabetic mice [230]. mPGES‐2 inhibitor SZ0232 suppresses β‐cell senescence via the PGE2–EP3–NR4A1 axis, enhancing insulin secretion and improving glycemia [210]. NAD^+^ supplementation restores mitochondrial calcium homeostasis and prevents diabetic peripheral neuropathy [231]. Caloric restriction (CR) enhances β‐cell autophagy and mitochondrial function, improves senescence markers, and increases insulin sensitivity [232].
Neurodegenerative diseases	p16↑ [182], p21↑ [233]	Senescent BAMs propagate senescence signals to brain parenchyma via migrasomes, amplifying neuroinflammation [187]. cGAS–STING activation sustains chronic type I interferon (IFN‐I) responses and neurotoxicity [186].	TSPAN4 siRNA blocks migrasome generation in BAMs, curtails senescence signal propagation, and reduces Aβ deposition [187]. D+Q attenuates neuroinflammation, lowers Aβ burden, and improves cognitive deficits [233]. NAD^+^ supplementation ameliorates Aβ pathology and neuroinflammation [234]. Young donor gut microbiota transplantation reverses immune aging and neuroinflammation [235].
Bone diseases	p16↑, IL‐6↑, TNF‐α↑, MMPs↑	Senescence of osteoblasts, osteocytes, and bone marrow stromal cells impairs bone formation and disrupts the resorption–formation balance [190]. Macrophage senescence drives OA pathology [193]. Sarcopenic fibroblasts sustain a SASP‐rich, fibrotic microenvironment [194].	pCQ/SOD nanoparticles suppress macrophage senescence and alleviate OA [193]. EM–eNMs restore mitochondrial homeostasis, reverse stem cell senescence, and attenuate bone loss [236]. miR‐29 targeting ameliorates mesenchymal stromal cell senescence and skeletal aging [237].

The overarching pattern is one in which telomere attrition and metabolic reprogramming converge on alveolar epithelial senescence and mechanotransduction failure, yielding ECM remodeling, progressive fibrosis, and a protumorigenic pulmonary microenvironment.

### 3.3 Liver Diseases

The aging liver accumulates senescent cells, sustains chronic inflammation, develops progressive fibrosis, and loses metabolic and detoxification capacity [167]. In metabolic dysfunction‐associated steatohepatitis (MASH), neutrophil extracellular traps (NETs) induce hepatocyte senescence that in turn propagates steatosis and fibrosis [168], and in metabolic dysfunction‐associated steatotic liver disease (MASLD), senescent hepatocyte burden correlates directly with the severity of metabolic disruption and fibrosis [169, 170, 171]. Activation of the cholesterol synthesis transcription factor SREBP2 drives hepatocyte senescence and impairs liver function [167]. Beyond their local effects, senescent hepatocytes actively shape the metabolic microenvironment: together with senescent macrophages, they accumulate the lipid peroxidation product 13‐HODE, which inhibits catalase activity and promotes age‐associated hepatic steatosis [172]. Through TGF‐β paracrine signaling, senescent hepatocytes can further propagate senescence to distant organs, linking hepatic aging to systemic multiorgan dysfunction [173].

Senescent cells in the liver, however, exhibit a context‐dependent duality. SASP factors can promote in vivo reprogramming and tissue regeneration in mice [174], and cGAS–STING–IRF3‐mediated senescence of hepatic stellate cells (HSCs) actually attenuates liver fibrosis [175]. In hepatocellular carcinoma (HCC), this duality is strikingly illustrated by FBP1: high FBP1 expression induces tumor‐suppressive hepatocyte senescence that restrains proliferation, but NRF2/AKT‐driven FBP1 degradation reverses this arrest, licensing precursor cells to re‐enter the cell cycle and drive HCC progression [176]. Simultaneously, FBP1‐deficient hepatocytes release HMGB1, inducing HSC senescence whose SASP then constructs a protumorigenic microenvironment that accelerates tumor growth [177]. This FBP1 axis crystallizes a fundamental paradox in liver senescence biology: cell‐autonomous hepatocyte senescence acts as a barrier to tumorigenesis, while noncell‐autonomous HSC senescence drives it. Upstream metabolic disturbances and aging signals thus converge on a hepatic senescence landscape whose consequences, including steatosis, fibrosis, or HCC progression, are ultimately determined by cellular context.

### 3.4 Neurodegenerative Diseases

Brain aging drives neurodegenerative pathology through multilevel molecular disruption, underpinning the onset and progression of AD, Parkinson's disease (PD), and Huntington's disease (HD). Structurally, blood–brain barrier integrity erodes, synaptic plasticity declines, and meningeal lymphatic drainage diminishes, collectively impairing metabolic waste and pathological protein clearance, fostering aggregation, and promoting cortical dysfunction and memory deficits [178, 179, 180, 181]. In AD, this manifests as Aβ deposition from impaired clearance and neurofibrillary tangle formation from tau hyperphosphorylation [178]. In PD, age‐related autophagic decline permits α‐Syn aggregation, while senescent astrocytes secrete proinflammatory factors that accelerate dopaminergic neuron death [182, 183]. In HD, senescence induced by mutant huntingtin‐driven DNA damage and mitochondrial dysfunction is simultaneously a pathological consequence and an amplifying driver, forming a self‐reinforcing neurodegenerative loop [184].

Microglial biology is central to this picture. Aging microglia undergo extensive transcriptional and epigenetic reprogramming that blunts phagocytic function, accelerating Aβ aggregation or triggering disproportionate inflammatory responses to α‐Syn [185]. Aged microglial mtDNA further engages cGAS–STING signaling, contributing to brain injury and cognitive decline [186]. Border‐associated macrophages (BAMs) are early participants in brain aging: intracellular Aβ40 accumulation drives them toward a senescent phenotype, and these senescent BAMs transmit senescence signals to the parenchyma via migrasomes, inducing paracrine microglial senescence and amplifying the neuroinflammatory cascade [187]. Therapeutic strategies targeting brain aging, therefore, focus on disrupting this inflammation‐degeneration cycle by clearing senescent cells and blocking senescence propagation, with demonstrated improvements in cognitive function (Table 1). In aggregate, upstream proteostasis failure, mitochondrial dysfunction, DNA damage, and impaired clearance converge on glial senescence as a central amplifier—driving pathological protein aggregation, neuronal loss, and cognitive decline across AD, PD, and HD.

### 3.5 Skeletal Diseases

Musculoskeletal decline is among the most clinically visible features of aging, manifesting as sarcopenia, OP, and OA. In skeletal muscle, senescent stem cells and myofibers accumulate oxidative damage and adopt a senescent state whose SASP factors suppress muscle regeneration through paracrine signaling and promote protein degradation, directly causing atrophy and functional loss [6, 188]. In aging bone, osteoblasts, osteocytes, and bone marrow stromal cells display functional impairment and upregulated senescence markers, including IL‐6 and matrix metalloproteinases (MMPs) [189, 190]. Within the local bone microenvironment, SASP factors simultaneously impair osteoblast function and promote osteoclast precursor survival and differentiation, partly through PI3K/Akt/NF‐κB signaling, tipping the balance toward net resorption and driving OP [191, 192]. In OA, senescent macrophages accumulate mitochondrial damage, and ROS buildup is a key driver of both mitochondrial dysfunction and the senescent phenotype [193]. In sarcopenia, fibroblast expansion generates an inflammatory and fibrotic environment that disrupts metabolic homeostasis and impedes muscle regeneration [194]. Across these conditions, oxidative stress and mitochondrial damage drive senescence in muscle stem cells, osteoblasts, and macrophages, whose SASP factors then erode tissue homeostasis, suppressing regeneration, promoting bone resorption, and inducing fibrosis, culminating in sarcopenia, OP, and OA.

### 3.6 Metabolic Diseases

Metabolic syndrome, comprising central obesity, impaired glucose metabolism, hypertension, and dyslipidemia, increases markedly in prevalence with age and can be considered the metabolic phenotype of systemic aging. Its core features, insulin resistance and chronic low‐grade inflammation, provide the shared pathological substrate for type 2 diabetes (T2D) and CVD [195, 196, 197, 198]. In aging adipose tissue, adipocyte hypertrophy is accompanied by macrophage and T cell infiltration, a process that may represent a defining feature of age‐related immune remodeling [199]. Macrophages recruited to hypertrophic, hypoxic adipose depots polarize toward proinflammatory phenotypes and are dominant mediators of systemic insulin resistance [200, 201]. When this inflammaging state reaches the vasculature, it lays the cellular groundwork for CVD: metabolic stress induces endothelial senescence, impairing NO synthesis, upregulating adhesion molecule expression, and facilitating monocyte infiltration and atherosclerotic plaque formation [202, 203, 204]. Senescent foam macrophages within plaques degrade the fibrous cap through MMP secretion, compounding plaque vulnerability and rupture risk [205]. Clinically, the IL‐6 inhibitor ziltivekimab has significantly reduced inflammatory biomarker levels in high‐risk atherosclerosis patients, providing direct validation for SASP‐targeting cardiovascular strategies [206].

At the pancreatic level, metabolic stress accelerates β‐cell senescence [207]. Senescent adipocytes worsen systemic insulin resistance and chronic inflammation while hastening compensatory β‐cell exhaustion [208, 209]. Senescent β‐cells exhibit impaired insulin secretion, and their SASP amplifies local islet inflammation and acts systemically on peripheral tissues, contributing to diabetic complications, including impaired wound healing [210, 211, 212, 213]. Human islet studies confirm that senescent β‐cell burden rises with age and diabetes duration, supporting a causal role in T2D progression [207]. Senescence in adipocytes, endothelial cells, and β‐cells thus functions as a shared hub through which metabolic stress and chronic inflammation are transduced into insulin resistance, atherosclerosis, and β‐cell failure, the cardinal pathologies of metabolic syndrome, T2D, and CVD, with aging‐targeted interventions demonstrating broad benefit across these endpoints in preclinical models (Table 1).

**FIGURE 3:**
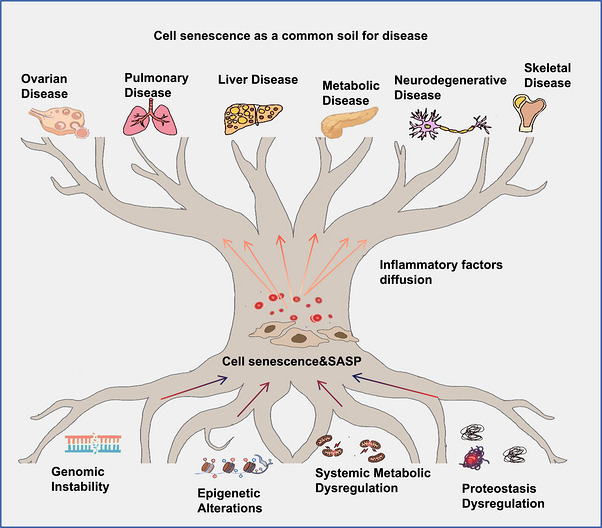
The common basis: shared molecular origins of divergent organ pathologies. This schematic traces the mechanistic convergence through which distinct organ pathologies emerge from a shared biological foundation. At the base, four interconnected molecular hallmarks form the roots of aging: genomic instability (DNA strand breaks and telomere attrition), epigenetic alterations (nucleosome disruption and histone modification imbalances), systemic metabolic dysregulation (ROS‐leaking, damaged organelles), and proteostasis loss (accumulation of toxic protein aggregates and impaired clearance). These upstream insults converge at the cellular level to drive senescence, the central conduit of pathology, depicted as a trunk through which senescent cells secrete SASP factors, including IL‐6 and TGF‐β, that propagate systemic inflammation and tissue remodeling. These toxic signaling cascades upward, producing divergent clinical phenotypes across the reproductive, pulmonary, hepatic, neurological, skeletal, and metabolic systems. The hierarchical structure of the figure reinforces the key therapeutic implication: targeting the molecular roots or the senescent cell hub offers a unified strategy for multimorbidity.

Across conditions as disparate as ovarian failure and bone disease, the molecular hallmarks of aging constitute a shared pathological foundation. These six diseases converge on a common mechanistic substrate (Figure 3): senescence in functionally critical cell populations and SASP‐driven chronic sterile inflammation. This convergence offers a compelling explanation for why organismal aging functions as a universal risk factor across otherwise distinct disease entities.

This shared foundation has elevated targeted senescent cell clearance to a broad‐spectrum therapeutic strategy of considerable promise. In IPF patients, lung senescence marker expression correlates with disease severity, and the first human senolytic trial demonstrated that D+Q combination therapy improves physical function in this population [238]. Subsequent studies extended these findings, showing that the same strategy reduces senescent cell burden in diabetic kidney disease and measurably lowers senescent cell load in adipose tissue and skin [239]. The growing pipeline of antiaging interventions entering clinical trials collectively underscores the translational momentum of this field. Beyond senescent cell elimination, strategies that restore cellular function rather than remove cells, including exercise and dietary interventions, have also yielded encouraging results and are examined in the following section.

## 4 Antiaging Strategies

**FIGURE 4:**
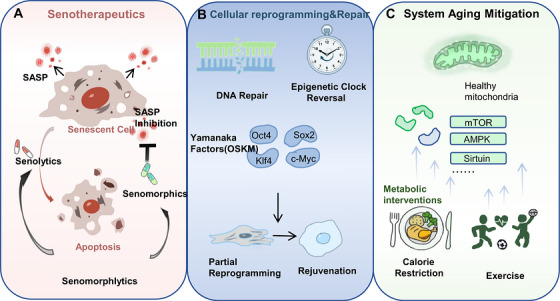
Multifaceted intervention strategies targeting the core hallmarks of aging. (A) Senotherapeutics: strategies that eliminate or modulate senescent cells characterized by irreversible cell cycle arrest and SASP secretion. (i) Senolytics: agents that selectively induce apoptosis in senescent cells. (ii) Senomorphics: agents that suppress the pathological SASP phenotype without eliminating the cell. (iii) Senomorphlytics: a novel dual‐action class integrating both clearance and phenotypic modulation. (B) Cellular reprogramming and repair: interventions that restore intrinsic cellular youthfulness and repair capacity. Partial reprogramming through transient OSKM expression resets the epigenetic clock and reverses age‐associated transcriptomic changes without altering cell identity, restoring youthful cellular morphology. Complementary enhancement of DNA repair mechanisms and TERT reactivation counteracts genomic instability and telomere attrition, thereby further supporting tissue regeneration. (C) Metabolic interventions: systemic strategies that remodel the aging microenvironment through metabolic reprogramming. Caloric restriction (CR) and exercise function as physiological stressors that suppress mTOR while activating AMPK and Sirtuins, collectively enhancing mitochondrial biogenesis and mitophagy, reducing oxidative stress, and restoring proteostasis to extend healthspan.

Aging research has advanced from phenomenological description to mechanistic dissection at the cellular, metabolic, genetic, and environmental levels, and this convergence of fundamental insights is now yielding actionable intervention strategies. Building on the core mechanisms detailed above, genomic instability, epigenetic drift, metabolic dysfunction, and proteostasis failure, current approaches fall into three broad directions: targeting senescent cells and their secretome; rejuvenating cellular function; and remodeling the aging environment through metabolic reprogramming (Figure 4). This section systematically evaluates the evidence for each strategy in preclinical and human studies and examines the opportunities and obstacles facing clinical translation.

### 4.1 Senotherapeutics

Through the SASP, senescent cells constitutively release proinflammatory cytokines, chemokines, and proteases that drive local and systemic inflammaging, disrupt the tissue microenvironment, impair neighboring cell function, and underpin a wide range of age‐related diseases [240].

#### 4.1.1 Senolytics

Senolytics selectively eliminate senescent cells to relieve age‐related tissue dysfunction and have become a leading antiaging strategy, spanning kinase inhibitors, Bcl‐2 family inhibitors, natural polyphenols, heat shock protein inhibitors, and antibiotics. The dasatinib‐plus‐quercetin (D+Q) combination, the first senolytic regimen to enter clinical trials, has reduced SASP factors and improved tissue function in IPF and diabetic kidney disease (Table 2). In HCC models, cotreatment with the BCL‐2 inhibitor ABT‐263 (navitoclax) and GCN2iB induces tumor cell apoptosis and promotes in vivo tumor regression [241]. BTSA1 selectively clears senescent myofibroblasts through BAX activation, effectively treating bleomycin‐induced pulmonary fibrosis [221], while the PROTAC‐based agent 753b reduces hepatic senescent cell burden in mice and suppresses MASLD progression to MASH and fibrosis [227].

**TABLE 2:** Clinical evidence for senescence‐targeted and antiaging interventions.

Intervention strategy	Intervention/agent	Trial ID	Population	Key findings	Study status	Phase	References
Senolytics	D+Q	NCT05422885	Older adults at high risk for AD	Safety: feasible, high adherence; biomarker: ↓TNF‐α; function: no significant overall change	Completed	Phase 1 (pilot)	[290]
	D+Q	NCT04685590	Amnestic mild cognitive impairment or early AD	N/A	Active, not recruiting	Phase 2	N/A
	D+Q	NCT04063124	Early symptomatic AD	Safety: well tolerated, 100% adherence, no SAEs; biomarker: ↑CSF IL‐6/GFAP, trend ↑Aβ42, ↓plasma SASP factors; function: no significant cognitive or imaging changes	Completed	Phase 1	[291]
	D+Q	NCT04313634	Postmenopausal women	Safety: no SAEs; biomarker: primary endpoint (CTx) not met; transient ↑P1NP at 2–4 weeks, not sustained; function: no overall BMD change	Completed	Phase 2	[292]
	D+Q	NCT02874989	IPF patients	Safety: well‐tolerated.	Completed	Phase 1	[293]
	UBX1325	NCT04537884	Advanced diabetic macular edema	Safety: well tolerated; function: improved visual acuity (≥6 months)	Completed	Phase 1	[294]
	PD‐1 inhibitor tislelizumab combined with D+Q	NCT05724329	Head and neck squamous cell carcinoma	Safety: favorable tolerability; biomarker: reversal of immunosenescence markers; function: major pathological response 33.3 vs. 7–13.9% with PD‐1 inhibitor alone	Active, not recruiting	Phase 2	[295]
	Cyclophosphamide, fludarabine, and chimeric antigen receptor (CAR)‐T cell therapy combined with D+Q	NCT06940297	Multiple myeloma	N/A	Recruiting	Phase 2	N/A
Exosomes	ahaMSCs‐Exos	NCT04388982	AD patients	Safety: well tolerated, no AEs; biomarker: no significant change in amyloid/tau deposition; function: medium‐dose improved cognitive function	Completed	Phase 1/2	[296]
Stem cells	Lomecel‐B	NCT05233774	Mild AD	Safety: primary endpoint met, well tolerated; biomarker: ↓whole brain volume decline 48.4%, ↓hippocampal decline 61.9%, ↓neuroinflammation; function: composite AD score improvement trend	Completed	Phase 2	[297]
Metabolic modulation and nutritional interventions	GlyNAC (glycine + N‐acetylcysteine)	NCT01870193	Older adults	Safety: well tolerated; biomarker: corrected glutathione deficiency and aging hallmarks; function: ↑muscle strength, gait speed	Completed	Early Phase 1	[298]
	Metformin	NCT03107884	Healthy older adults	Biomarker: ↓muscle and fibro‐adipogenic progenitor senescence/SASP; function: ↑type I myofiber cross‐sectional area, ↓fibrosis	Active, not recruiting	Early Phase 1	[299]
	Nucleotide	NCT05243108	Older adults	Safety: no SAEs, well tolerated; biomarker: ↓DNA methylation age, no significant change in telomere length; FUNCTION: ↑insulin sensitivity	Completed	N/A	[300]
	Nicotinamide riboside (NR)	NCT04990869	COPD patients	Safety: no SAEs, well tolerated; biomarker: ↓sputum IL‐8, ↑blood NAD^+^	Completed	N/A	[223]
	Protein‐ and prebiotic‐containing supplement	NCT04309292	Older adults	Safety: well tolerated; biomarker: ↑gut bifidobacterium; function: ↑cognition; no significant change in muscle strength or physical function	Completed	N/A	[301]
	Intermittent fasting and healthy lifestyle guidance	NCT02460783	Overweight older adults with normal cognition and insulin resistance	Safety: well tolerated; biomarker: ↓neuronal insulin resistance markers, ↓BrainAGE, ↓brain glucose, ↓metabolic parameters; no significant change in AD biomarkers; function: ↑executive function and memory	Completed	N/A	[302]
	Polyphenol‐rich diet	NCT03020186	Abdominal obesity or dyslipidemia	Biomarker: ↓epigenetic age (Li/Hannum clocks) with Green‐MED diet	Completed	N/A	[303]
	Vitamin D, Omega‐3, and exercise	NCT01745263	Healthy older adults	Biomarker: omega‐3 ↓PhenoAge, GrimAge2, DunedinPACE; additive triple benefit on PhenoAge	Completed	Phase 3	[304]

Synthetic biology has introduced a new dimension to senolytic therapy. CAR‐T cells engineered to recognize the senescence marker urokinase‐type plasminogen activator receptor (uPAR) clear senescent cells potently and durably from a single administration, providing proof‐of‐concept for a one‐dose, long‐lasting antiaging strategy. The synthetic peptide FOXO4–DRI selectively triggers apoptosis in senescent cells by disrupting the FOXO4–p53 interaction, promoting nuclear p53 exclusion and restoring its proapoptotic activity; structural analyses confirm that FOXO4–DRI binds directly to the p53 transactivation domain 2 (TAD2), with p53 phosphorylation strengthening this interaction [242]. In models of bleomycin‐induced pulmonary fibrosis and keloids, FOXO4–DRI markedly reduced senescent cell burden and improved tissue pathology, demonstrating translational potential for antifibrotic applications [243, 244].

#### 4.1.2 Senomorphics

Rather than eliminating senescent cells, senomorphics modulate their phenotype and secretory behavior to blunt pathological effects [245]. Natural compounds have attracted particular interest for their multitarget activity: rutin suppresses proinflammatory mediator release via the ATM–TRAF6/HIF1α axis and exhibits synergistic antitumor effects with chemotherapy in immunodeficient mice [246]; HK, derived from Bolivian prawn sage (*Salvia haenkei*) with luteolin as its principal active component, disrupts the p16–CDK6 interaction and attenuates fibrosis and inflammation in naturally aged mice [247]; and a niacinamide–hyaluronic acid combination has shown promising antiaging activity in skin [248].

#### 4.1.3 Senomorphlytics

Senomorphlytics integrate senolytic and senomorphic mechanisms to balance efficacy with safety [249]. A triple combination of quercetin, β‐sitosterol, and salicylic acid reverses multitissue aging phenotypes in mammals [249]. A bifunctional nanozyme exploits dose‐dependent duality: at high doses, it selectively induces cuproptosis and ferroptosis in senescent cells, while at low doses, it remodels heterochromatin and suppresses SASP gene expression, producing therapeutic benefit in atherosclerosis models [250]. The natural compound procyanidin C1 (PCC1) reverses structural and functional retinal aging by simultaneously modulating BCL‐2 family proteins and impairing mitochondrial function to induce senescent cell apoptosis, while directly blocking upstream inflammatory signaling to restore visual function [251]. Collectively, these advances reinforce the centrality of senescent cells in organ aging and disease.

#### 4.1.4 Milestones in the Evolution of Senotherapeutics

**FIGURE 5:**
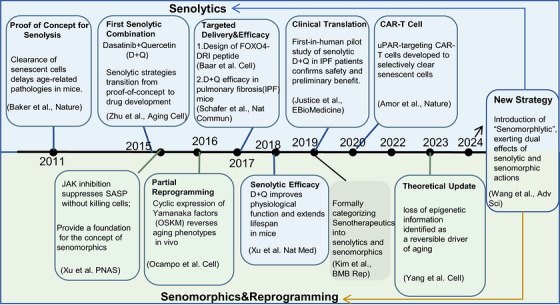
Timeline of pharmacological and genetic interventions targeting senescence. This timeline traces the rapid evolution of senotherapeutic strategies over the past 15 years, from initial proof‐of‐concept in transgenic mouse models to small‐molecule drug discovery and early clinical translation. The upper panel highlights senolytic strategies targeting the elimination of senescent cells, including D+Q, FOXO4‐DRI, and CAR‐T cell approaches. The lower panel covers senomorphic and reprogramming strategies aimed at SASP modulation or cellular age reversal, including JAK inhibitors and expression of the Yamanaka factors. The timeline culminates in 2024 with the emergence of senomorphlytics as a dual‐action therapeutic paradigm. *Abbreviations*: D+Q, dasatinib and quercetin; SASP, senescence‐associated secretory phenotype; OSKM, Oct4, Sox2, Klf4, and c‐Myc; IPF, idiopathic pulmonary fibrosis; CAR‐T, chimeric antigen receptor T cell.

The senotherapeutics field has evolved rapidly over the past 15 years. The conceptual foundation was laid in 2011, when Baker et al. demonstrated in the INK–ATTAC mouse model that senescent cell clearance delays age‐related pathologies [6]. In 2015, Zhu et al. identified D+Q as the first small‐molecule senolytic combination, marking the field's transition from concept to drug development [252], while Xu et al. showed that JAK pathway inhibition suppresses SASP without eliminating senescent cells, thereby alleviating systemic inflammation and frailty in aged mice [253], establishing senomorphics as a complementary phenotype‐modulating strategy [254]. A pivotal conceptual shift arrived in 2016, when Ocampo et al. showed that transient, cyclic Yamanaka factor expression reverses aging phenotypes across multiple tissues in a premature aging mouse model without inducing tumorigenesis, opening a new frontier in epigenetic rejuvenation [255]. In 2017, Baar et al. introduced FOXO4–DRI as a senescent cell‐targeting peptide [256], and Schafer et al. demonstrated that D+Q selectively clears senescent cells in IPF mice while improving lung function and physical performance [159]; subsequent studies in aged mice confirmed that D+Q also extends lifespan [257]. These advances culminated in the first human senolytic trial: in 2019, Justice et al. reported that D+Q was safe and yielded preliminary signals of clinical benefit in IPF patients [238]. In the following years, Amor et al. engineered uPAR‐targeting CAR‐T cells for senescent cell clearance, demonstrating the antiaging potential of synthetic biology [258]. In 2023, Yang et al. established that epigenetic information loss is a reversible driver of senescence, forging a mechanistic link between DNA damage and epigenetic aging and providing the theoretical grounding for partial reprogramming [259]. In 2024, the dual‐mechanism compound JM10101 exemplified the emerging senomorphlytic paradigm [249]. The clinical landscape for antiaging interventions continues to broaden well beyond senolytics (Figure 5).

The inclusion of partial reprogramming in this timeline signals a fundamental paradigm shift, from treating or clearing aged cells to restoring their intrinsic youthfulness. This emerging direction moves the therapeutic focus from external intervention to endogenous rejuvenation, as discussed in the following section.

### 4.2 Rejuvenating Cellular Functions

Framing senescent cells purely as pathological waste to be eliminated risks overlooking their constructive roles in tissue repair and embryonic development. Preclinical data also indicate that some senolytics carry meaningful side effects, notably thrombocytopenia, which complicate their long‐term use [260]. A complementary and arguably more fundamental strategy, therefore, lies in bolstering the cell's own repair and regenerative machinery, thereby addressing aging at its source rather than managing its cellular output.

#### 4.2.1 Partial Reprogramming

Partial reprogramming leverages iPSC technology by transiently expressing Yamanaka factors (OSKM or OSK) to reverse senescence without permanently altering cell identity [255]. In both human cells and mouse models, this approach resets the epigenetic clock, reduces inflammation, restores mitochondrial function, and restores regenerative capacity in aged stem cells [259, 261, 262, 263]. A key mechanistic insight has recently emerged: aging is accompanied by widespread loss of cell identity, termed mesenchymal drift, and partial reprogramming exerts its primary effect by preferentially reversing this drift, producing multilayered improvements in cellular aging phenotypes [264]. This finding directly supports epigenetic dysregulation as a core aging driver and delineates the principal pathway through which partial reprogramming operates, strengthening its mechanistic foundation as a therapeutic strategy.

#### 4.2.2 Telomerase Reverse Transcriptase Activation and DNA Repair Enhancement

Inadequate telomerase activity arising from low telomerase reverse transcriptase (TERT) transcription is a central contributor to cellular senescence. The small molecule TAC restores TERT levels in aged tissues, reducing senescence and systemic inflammation, promoting neurogenesis, and improving cognitive and muscle function in very old mice, providing preclinical proof‐of‐concept for TERT‐activating strategies [265]. The circular RNA circHERC1 acts as a positive regulator of TERT transcription, preserving telomere integrity and delaying senescence [266]. Equally important, the age‐associated decline in DNA repair capacity, manifested as widespread downregulation of repair genes in senescent cells [267], can be partially offset through intercellular cooperation: apoptotic vesicles transfer nuclear DNA repair enzymes, including PARP1, to neighboring cells, repairing their DNA damage and suppressing premature senescence [268], revealing an endogenous cooperative repair mechanism with therapeutic implications.

#### 4.2.3 Exosome and Stem Cell Therapies

The concept of senoreverse, actively reversing rather than eliminating senescence, has gained traction through several innovative delivery approaches. Exosome‐mediated transfer of miR‐302b selectively suppresses the cell cycle inhibitors *Cdkn1a* and *Ccng2*, reactivating proliferative capacity in previously senescent cells and reversing multiple aging phenotypes, offering a transformative direction for age‐related disease therapy [269]. Small extracellular vesicles from human amniotic mesenchymal stem cells (hAMSC‐sEVs), enriched in miR‐21‐5p, target IL‐6RA and block STAT3 phosphorylation and nuclear translocation; this relieves STAT3‐mediated transcriptional repression of the mitochondrial calcium uniporter, epigenetically resetting the β‐cell senescence program and restoring mitochondrial homeostasis and insulin secretion [270]. Senescence‐resistant human mesenchymal progenitor cells engineered through synthetic biology have demonstrated broad systemic benefits in primate models, including limiting multiorgan aging, enhancing cognition, and reducing whole‐body senescent cell accumulation, with exosome release identified as a partial mediator of efficacy, establishing a new paradigm for cell‐based systemic antiaging intervention [271].

### 4.3 Remodeling Systemic Metabolism

Metabolic reprogramming addresses aging systemically by modulating nutrient‐sensing pathways and restoring mitochondrial function, offering a whole‐organism intervention route rather than a cell‐type‐specific one.

#### 4.3.1 CR and Its Mimetics

CR, reduced total caloric intake without malnutrition, is the most reproducible lifespan‐extending intervention identified across species, from yeast to nonhuman primates [272]. In humans, CR demonstrably improves aging‐associated metabolic complications, including obesity, insulin resistance, muscle loss, and dyslipidemia, without compromising quality of life [273]. Its broad efficacy reflects the ability to remodel systemic physiological networks through convergent effects on nutrient‐sensing pathways [274]: mTOR, AMPK, and Sirtuin signaling act synergistically to regulate mitochondrial metabolism, oxidative stress, inflammation, and the gut microbiome [84, 275, 276]. Energy limitation during CR inhibits mTORC1, activates AMPK, promotes autophagy, enhances stress resistance, and restores metabolic homeostasis [89].

This mechanistic framework has guided the development of CR mimetics. LCA activates Sirtuin deacetylases and AMPK by targeting TULP3, replicating CR's lifespan‐extending effects across multiple species [277, 278]. Metformin activates AMPK via PEN2 through the lysosomal pathway [279]; rapamycin directly inhibits mTORC1 [85]; and resveratrol promotes autophagy through AMPK activation with broad antiaging effects in myocardial ischemia–reperfusion injury and UV‐induced photoaging models [280, 281, 282]. These agents partially replicate CR's effects and suppress diet‐induced aberrant liver proteome reprogramming [283]. Among them, rapamycin may more faithfully reproduce CR‐driven longevity benefits than metformin at the vertebrate level [284]. CR's antiaging potency ultimately reflects the integration of multiple mechanisms: modulation of β‐cell function and insulin sensitivity [232], suppression of systemic inflammation [275], and gut microbiome remodeling [276]. It bears noting that rapamycin and metformin are also classified as senomorphics, an overlap that illustrates how nutrient‐sensing dysregulation is itself a root driver of senescence and SASP secretion.

#### 4.3.2 Exercise

Exercise is among the most extensively validated strategies for metabolic homeostasis and healthy aging. Training shifts the epigenetic and transcriptomic profiles of aged muscle toward a more youthful state, while disuse accelerates transcriptomic aging [285]. Long‐term exercise reduces circulating inflammatory factors, stimulates renal betaine secretion, and suppresses innate immune kinase TBK1 activity to blunt inflammaging signaling [286]. Exercise elevates circulating levels of cardiotrophin‐like cytokine factor 1, a bone‐protective factor that declines with age, improving motor function, glucose tolerance, and mitochondrial function to counter musculoskeletal aging [287]. Beyond inflammation, exercise curbs epigenetic aging by downregulating immune mediators such as β2‐microglobulin and modulating immune cell function [288] and upregulates TERT activity to maintain telomere integrity and prevent senescence [289]. As a low‐cost, multitarget intervention operating across biological levels simultaneously, exercise represents one of the most potent and broadly accessible tools in the antiaging arsenal.

### 4.4 Clinical Translation: Progress and Challenges

#### 4.4.1 Current Landscape of Clinical Interventions

A growing number of antiaging strategies have entered clinical evaluation on the strength of preclinical evidence. As summarized in Table 2, current trials are shaped by regulatory frameworks that require targeting specific age‐related diseases, such as AD, IPF, and COPD, rather than aging per se. Leading interventions include repurposed drugs such as metformin and rapamycin, senolytics led by D+Q, and NAD^+^ precursors such as nicotinamide riboside (NR). Most trials remain at Phase I/II, with primary endpoints focused on safety and tolerability, yet encouraging signals have emerged for improvements in aging biomarkers and metabolic health. Translating mouse lifespan extension to human healthspan improvement, however, remains a formidable undertaking that demands rigorous validation over longer timeframes and more stringent endpoints.

#### 4.4.2 Limitations and Risks of Aging‐Targeted Interventions

Despite its momentum, the clinical translation of antiaging science faces substantial unresolved challenges. For most intervention strategies, the evidence chain remains anchored to preclinical models or surrogate endpoints, including epigenetic clocks, DNA methylation age, and inflammatory markers, with large‐scale, long‐term randomized controlled trials powered for hard endpoints such as delayed cognitive decline or reduced disease incidence still absent. Dosing, treatment duration, and the identification of reliable biomarkers represent further uncertainties that complicate trial design.

More fundamentally, questions of specificity and safety loom large. Senescent cells are not uniformly harmful: they contribute indispensably to wound healing, tissue repair, and tumor immune surveillance [305, 306], support neonatal cardiac regeneration, and drive limb regeneration in salamanders [307, 308]. Indiscriminate clearance carries real costs—ABT‐263 treatment has been linked to fibrotic scarring [307], and suppressing SASP via AAV9‐mediated Gata4 knockdown worsened cardiac function after myocardial infarction in mice [309], underscoring the context‐dependent, proregenerative value of the SASP that must not be sacrificed [310]. Compounding the specificity problem is the profound heterogeneity of senescent cells: their phenotype and functional consequences differ markedly by tissue of origin and inducing stimulus. A single mouse study crystallizes this point, clearing p16^Ink4a+^ macrophages alleviated hepatocyte injury in liver fibrosis, whereas clearing p16^Ink4a+^ endothelial cells exacerbated it [311].

At the regulatory and ethical levels, aging's status as a natural biological process rather than a recognized disease indication forces drug development toward specific age‐related multimorbidities, demanding ever‐greater precision in target selection [312]. The field must therefore shift from broad elimination toward context‐aware, precise regulation, strategies that maximize healthspan benefit while preserving the physiological functions of senescent cells. This precision imperative is the central prerequisite for translating aging science into safe, durable clinical gains.

## 5 Discussion and Conclusion

The unprecedented demographic shift toward an aging global population demands a fundamental reorientation of medicine away from reactive, disease‐by‐disease treatment and toward proactive strategies that address the biological roots of aging itself. As this review synthesizes, aging is not stochastic wear and tear but a systemic process governed by a deeply interconnected network of molecular hallmarks: genomic instability, epigenetic drift, mitochondrial dysfunction, and proteostasis collapse. Cellular senescence sits at the center of this network, functioning as the critical transducer that converts intracellular molecular damage into the extracellular SASP, cultivating the shared pathological soil from which pulmonary fibrosis, hepatic steatosis, neurodegeneration, and cardiometabolic disease all grow.

The translation of these mechanistic insights into therapeutic action marks the opening of geroscience's clinical era. Intervention strategies have evolved rapidly from broad senescent cell clearance (senolytics) and secretory phenotype modulation (senomorphics), to partial reprogramming and metabolic remodeling that restore cellular vitality systemically. Early clinical trials have delivered proof of concept, yet the inherent duality of senescence, its indispensable roles in tissue repair and immune surveillance, make it clear that the next generation of therapies must move beyond blanket elimination toward precision regulation.

Recent years have seen meaningful progress across assessment, intervention, and clinical translation. On the biomarker front, Suryadevara et al. and the SenNet working group have systematically reviewed tissue‐specific senescence markers and issued detection recommendations [313]. At the same time, Ogrodnik et al. and Wu et al. have proposed standardized research guidelines and comprehensive biomarker frameworks [314, 315]. Therapeutically, Letai et al. applied BH3 profiling to quantify BCL‐xL dependence in senescent cell mitochondria, enabling prediction of sensitivity to senolytics such as ABT‐263 or D+Q and advancing treatment individualization [316]. Magkouta et al. exploited the universal accumulation of lipofuscin in senescent cells to develop a nanoplatform (mGL392) that couples a lipofuscin‐binding domain with a senolytic payload, enabling selective clearance while sparing normal cells [317]. Clinically, the mesenchymal stem cell therapy Lomecel‐B demonstrated favorable safety in Phase II trials alongside signals of reduced brain atrophy and improved cognitive function [297]. Together, these advances push aging research toward a more quantifiable, predictable, and safe paradigm.

Scientific progress alone, however, cannot fulfill the promise of healthy aging. As interventions move from laboratory to clinic, equity of access becomes a moral imperative. Advanced therapies such as CAR‐T senolytic regimens or personalized reprogramming protocols carry costs that risk confining extended healthspan to the wealthy, a concern especially acute in low‐ and middle‐income countries (LMICs), where regulatory infrastructure, clinical trial capacity, cold‐chain logistics, and locally led aging research remain underdeveloped. The healthcare access framework of Levesque et al., encompassing approachability, acceptability, availability, affordability, and appropriateness, provides a useful lens for mapping these multidimensional barriers, which extend well beyond drug pricing [318].

Closing this global divide requires a deliberate, multipronged deployment strategy: prioritizing scalable, oral‐formulation platforms that bypass complex infrastructure; establishing technology transfer mechanisms modeled on precedents such as the WHO mRNA vaccine hub to enable LMIC‐based manufacturing; and embedding preventive geroprotection, anchored in accessible dietary and exercise interventions, within primary care systems. Without sustained investment in these translational and capacity‐building pathways, the healthspan revolution will deepen rather than narrow existing global health inequities.

Ultimately, realizing the potential of aging science demands a transdisciplinary synthesis that integrates molecular precision, clinical rigor, and social responsibility in equal measure. By targeting the shared molecular origins of age‐related diseases while simultaneously dismantling barriers to global access, we can work toward a future in which the extension of human healthspan is not merely a scientific achievement but a universal human right.

## Author Contributions

Guowei Cai: visualization and writing – original draft. Jia Ren: visualization and writing – original draft. Li Wang: visualization and writing – original draft. Yingying Wu: visualization and writing – original draft. Huirui Wang: visualization. Xiaomeng Cao: visualization. Hao Shang: visualization. Xuben Hou: writing – review and editing. Yujiu Wang: writing – review and editing. Haibo Xue: supervision and writing – review and editing. Ting Dong: methodology, supervision, and writing – review & editing. All authors have read and approved the final manuscript.

## Funding

The authors have nothing to report.

## Ethics Statement

The authors have nothing to report.

## Conflicts of Interest

The authors declare no conflicts of interest.

## Data Availability

The authors have nothing to report.
